# Pregnancy outcomes following in vitro fertilization treatment in women with previous recurrent ectopic pregnancy

**DOI:** 10.1371/journal.pone.0272949

**Published:** 2022-08-15

**Authors:** Yamei Xue, Xiaomei Tong, Haocheng Zhang, Songying Zhang

**Affiliations:** 1 Department of Obstetrics and Gynecology, Assisted Reproduction Unit, Sir Run Run Shaw Hospital, School of Medicine, Zhejiang University, Hangzhou, China; 2 Department of Obstetrics and Gynecology, Key Laboratory of Reproductive Dysfunction Management of Zhejiang Province, Hangzhou, China; Central laboratory, The Affiliated Yantai Yuhuangding Hospital of Qingdao University, CHINA

## Abstract

**Objectives:**

The aim of this study was to investigate the impact of a history of recurrent ectopic pregnancy (EP) on pregnancy outcomes of subsequent in vitro fertilization (IVF) treatment.

**Methods:**

A retrospective cohort study involving 457 women with a history of recurrent EP (REP group), 912 women with a history of single EP (SEP group), and 1169 women with a history of intrauterine pregnancy (IUP group) as the control group, was conducted. IVF outcomes were compared for each cohort.

**Results:**

The incidence of EP in the REP group after IVF treatment was significantly lower than those in the SEP group (2.4% vs. 6.8%, P = 0.011), and similar to those in the IUP group (2.4% vs. 2.1%, P = 0.830). No significant differences were observed in the clinical pregnancy rate, miscarriage rate, and live birth rate among the three groups. There was no statistically significant difference in the recurrent EP rate between the salpingectomy and salpingostomy treatments. Adjusting for maternal and treatment factors did not influence live birth rates for women with previous REP compared with women with previous SEP and those with IUP. The odds of EP were 82.2% lower (OR 0.178, 95% CI 0.042–0.762; P = 0.020) in women who had blastocyst transfer compared with cleavage embryo transfer in the SEP group. The odds of EP were over six times (OR 6.260, 95% CI 1.255–31.220; P = 0.025) in women who underwent double embryo transfer as opposed to single embryo transfer in the IUP group.

**Conclusion:**

Our results indicate that women with previous recurrent EP have a lower risk of EP after IVF in comparison with women with previous single EP. Previous EP has no significant adverse effect on the main IVF outcomes. The salpingostomy and salpingectomy treatments of EP do not significantly affect the incidence of recurrent EP after IVF.

## Introduction

Ectopic pregnancy (EP) which accounts for about 1–2% of all spontaneous pregnancies, is the most cause of maternal death during the first trimester of pregnancy [1–3]. With the wide application of assisted reproductive technologies (ART), the incidence of EP trends to increase, occurring in approximately 1.4–3.2% of pregnancies after IVF treatment [4–6].

Compared with patients with no history of EP, patients with a history of EP had a higher risk of recurrent EP after IVF treatment, which ranged from 0.6% to 8.9% in the published studies [7–9]. Previous studies assessing the pregnancy outcomes only focused on women with a previous single EP [7–10]. There are few data on pregnancy outcomes in women with previous recurrent EP who undergoing IVF treatment [11–14]. Moreover, most studies have focused on the risk factors for recurrent EP [11–13], or on optimizing treatment methods to preserve fertility [14]. Infertile patients, who have a history of recurrent EP, may be particularly worried about future reproductive outcomes including the pregnancy rate and the likelihood of EP after IVF treatment. However, there is little evidence available in the studies to guide physicians in counseling this specific group of women.

The aim of this present study was to investigate the impact of a history of single EP and recurrent EP on pregnancy outcomes of subsequent IVF treatment. We also evaluated the effect of the different EP treatments on the incidence of EP.

## Materials and methods

### Subjects

We performed this retrospective cohort study and collected electronic records of women who underwent IVF/ICSI treatments at the Reproductive Medicine Center, Sir Run Run Shaw Hospital between January 2016 and May 2020. The study was approved by the Reproductive Medical Ethics Committee of Sir Run Run Shaw Hospital, College of Medicine, Zhejiang University. The names of patients were not divulged, the requirement for informed consent was therefore waived.

Inclusion criteria were infertility women with (1) a history or histories of tubal EP treated by surgery, or intrauterine pregnancy (control group) from a natural pregnancy before IVF treatment, (2) regular menstrual cycle (interval 21–35 days), and (3) undergoing the first fresh or frozen embryo transfer cycle. The exclusion criteria were as follows: (1) the previous EP resulted from ART (IVF/ICSI and related technology), (2) patients with conservative treatment of previous EP, (3) oocyte donor cycles, (4) the cycles of preimplantation genetic diagnosis and screening; and (5) the cycles involving incomplete records. The control group with intrauterine pregnancy was matched to the experimental group using the criteria: (1) age (± 1 year); (2) body mass index; (3) the level of basal serum FSH; (4) presence of male factor infertility; and/or (5) presence of endometriosis or pelvic inflammatory disease. We required exact matching for criteria 1–3, and we attempted to match criteria 4–5 as closely as possible. During the study period, a total of 457 women with a history of recurrent EP (REP group), 912 women with a history of single EP (SEP group), and 1169 women with a history of intrauterine pregnancy (IUP group) as control group were analyzed. To evaluate the effect of the different EP treatments on the incidence of recurrent EP, we further divided into two groups: salpingostomy and salpingectomy treatments.

We collected baseline characteristics including maternal age, body mass index, infertility duration, basal serum FSH, LH, and E2 levels, antral follicle count (AFC), cause of infertility, stimulation protocol, duration of stimulation, parity, preexisting condition of pelvic inflammatory disease, methods of EP treatments (salpingectomy or salpingostomy). The evaluated parameters of IVF cycles included the number of oocytes retrieved, methods of fertilization, the normal fertilization rate, stage of embryos transferred, type of embryo transfer, and the number of embryos transferred.

### IVF procedure

As previously described [6], controlled ovarian hyperstimulation (COH) was performed to maximize follicular response while minimizing the risk of ovarian hyperstimulation syndrome. The dose of gonadotropin (Gonal-F, Serono Laboratories, Aubonne, Switzerland; or Puregon, N.V. Organon, Oss, the Netherlands) was individually adjusted according to female age, weight, day 3 serum FSH value, and antral follicle count. Human chorionic gonadotropin (hCG) (6500–10,000 IU; Serono Laboratories, Modugno, Italy) was administered in patients when three or more follicles reached 16–18 mm or more.

Transvaginal ultrasonography-guided oocyte retrieval was performed 35 to 37 hours after the administration of hCG. Conventional IVF or intracytoplasmic sperm injection (ICSI) was used for fertilization. Embryos were cultured individually in sequential media in microdrops under mineral oil. Embryonic development was assessed on day 3, day 5, or day 6 after oocyte retrieval. The fresh embryo transfer took place on day 3, day 5, or day 6 under ultrasound guidance. If the whole embryos were frozen, the thawed embryo transfer occurred in natural cycles or hormone replacement treatment cycles. The number of transferred embryos or blastocysts was based on the Fourth Session of the Committee of Chinese Society of Reproductive Medicine (CSRM) guideline [15]. Regardless of maternal age and number of transfer cycles, no more than two embryos transferred is recommended. Single ET is suggested when the patient is young and had more than one good-quality embryo.

The embryo(s) was/were transferred using a soft catheter (Sydney®, Cook, Melbourne, Australia) under transabdominal ultrasound guidance. Before ET, patients were asked to keep filling of the bladder to facilitate an ultrasound view of the uterine cavity. The catheter was loaded with embryo(s) in a volume of about 10 μl of transfer medium. The embryo(s) was/were replaced approximately 1–1.5 cm from the uterine fundus under ultrasound visualization. After transfer, the catheter was immediately and carefully checked for retained embryos.

### Outcomes

The definition of EP referred to a pregnancy when the fertilized ovum implants outside the uterine cavity. Heterotopic pregnancy was defined by the co-occurrence of ectopic pregnancy and intrauterine pregnancy. In this study, heterotopic pregnancy was also grouped into EP. Clinical pregnancy was defined as visualization of the gestational sac with fetal heartbeat by transvaginal ultrasound 35 days after embryo transfer. Live birth referred to the delivery of one or more live infants. The miscarriage was defined as spontaneous abortion or intrauterine demise before 24 weeks of gestational age. The EP and miscarriage rates were calculated as per the number of clinical pregnancies.

### Statistical analysis

The statistical analysis was conducted in Statistical Package for Social Sciences version 20.0 (SPSS, Chicago, IL, USA). For continuous variables, we presented mean and standard deviation (SD) for symmetrical distribution or median and range (minimum-maximum values) for asymmetrical distributions. The variables were compared with one way ANOVA test or the nonparametric Kruskal-Wallis test depending on whether the data showed a normal distribution. Categorical variables were compared with Pearson’s Chi-squared or Fisher’s exact test based on sample size. Binary logistic regression was used to identify the odds ratio (OR) and 95% confidence interval (CI) for factors independently related to reproductive outcomes. Variables that are believed to influence both histories of ectopic pregnancy and pregnancy outcome were considered potential confounders [5, 9, 10]. These variables included female age, infertility duration, antral follicle count, cause of infertility, stimulation protocols, duration of stimulation, the number of oocytes retrieved, fertilization rate, stage of embryos transferred, type of embryos transferred, and the number of embryos transferred. The result was considered significant if the P-value was <0.05.

## Results

The characteristics of patients are presented in Table 1. No significant differences were observed in maternal age, BMI, basal serum FSH, LH and E2 levels, parity, and prevalence of the pelvic inflammatory disease among the groups. The average duration of infertility was significantly longer for women with SEP compared with women with previous REP or IUP (SEP vs. REP: 3.7 ± 3.1 vs.3.4 ± 2.9, P = 0.006; SEP vs. IUP: 3.7 ± 3.1 vs. 3.3 ± 2.7, P < 0.001). Women in IUP group had a significantly higher AFC than those with SEP and REP (IUP vs. SEP: 7.8 ± 4.0 vs. 8.2 ± 3.7, P < 0.001; IUP vs. REP: 8.2 ± 3.7 vs. 7.8 ± 4.2, P < 0.001). In addition, the percentage of tubal factor infertility was significantly higher in SEP and REP groups than in the control group. There was a greater proportion of couples with male factor infertility in the IUP group compared with couples in SEP and REP groups. The infertility factors of ovulatory disorder and endometriosis were similar among the three groups of patients. No statistically significant difference in the proportion of ovarian stimulation protocols among the three groups was observed. The duration of stimulation was significantly shorter in the REP group than in the IUP group (8.4 ± 2.6 vs. 8.7 ± 2.7, P = 0.030). Table 2 shows that women with previous REP had a lower median number of oocytes retrieved (7; range,1–34 vs 8; range, 1–36; P = 0.002) than those in the control group. The percentage of blastocyst transfer in the IUP group was significantly higher than that in the SEP group (32.2% vs. 26.3%, P = 0.004). There was a higher proportion of frozen-thawed cycles in the SEP group compared with those in the IUP group (72.6% vs. 68.0%, P = 0.024). The percentage of double embryo transfer in the IUP group was significantly lower than that in the SEP group (57.1% vs. 65.8%, P < 0.001).

**Table 1:** Characteristics of patients in the three groups.

	SEP	REP	IUP	*P*-value ^a^		
	(n = 912)	(n = 457)	(n = 1169)			
				P1	P2	P3
Female age	32.9 ± 4.8	32.3 ± 4.8	32.7 ± 4.7	0.080	0.641	0.149
< 30 (%)	252 (27.6)	139 (30.4)	313 (26.8)			
30 to < 35 (%)	348 (38.2)	170 (37.2)	475 (40.6)			
≥ 35 (%)	312 (34.2)	148 (32.4)	381 (32.6)			
BMI, kg/m^2^	21.2 ± 2.4	21.2 ± 2.3	21.3 ± 2.3	0.775	0.235	0.160
Infertility duration, y	3.7 ± 3.1	3.4 ± 2.9	3.3 ± 2.7	0.006	0.000	0.949
Basal serum FSH, IU/L	8.0 ± 3.0	7.9 ± 2.6	7.8 ± 2.9	0.457	0.220	0.082
Basal serum LH, IU/L	4.7 ± 2.7	4.4 ± 2.8	4.8 ± 3.5	0.761	0.556	0.229
Basal serum E2, ng/L	37.9 ± 22.9	38.2 ± 28.3	39.3 ± 26.5	0.141	0.784	0.099
AFC, n	7.8 ± 4.0	7.8 ± 4.2	8.2 ± 3.7	0.695	0.000	0.000
Cause of infertility (%)						
Tubal factor	912 (100)	457(100)	641 (54.8)	1.000	0.000	0.000
Ovulatory disorder	96 (10.5)	48 (9.9)	127 (10.9)	0.990	0.805	0.833
Endometriosis	82 (9.0)	35 (7.2)	108 (9.2)	0.406	0.846	0.312
Male factor	166 (18.2)	103 (21.1)	341(29.2)	0.057	0.000	0.007
Stimulation protocol (%)				0.377	0.105	0.369
Long protocol	462 (50.7)	233 (51.0)	632 (54.1)			
Short protocol	178 (19.5)	76 (16.6)	208 (17.8)			
Micro-stimulation protocol	157 (17.2)	93 (20.4)	215 (18.4)			
Others	115 (12.6)	55 (12.0)	114 (9.8)			
Duration of stimulation, d	8.6 ± 2.6	8.4 ± 2.6	8.7 ± 2.7	0.243	0.188	0.030
Parity (%)						
0	771 (84.5)	374 (81.8)	965 (82.5)	0.203	0.226	0.735
≥ 1	141 (15.5)	83 (18.2)	204 (17.5)			
PID (%)	107 (11.7)	47 (10.3)	148 (12.7)	0.424	0.522	0.185

*Note*: Values as mean ± standard deviation or number (%).

^a^We defined the statistical outcomes of SEP group versus REP group as P1; SEP group versus IUP group as P2; REP group versus IUP group as P3.

Abbreviations: SEP, single ectopic pregnancy; REP, recurrent ectopic pregnancy; IUP, intrauterine pregnancy; BMI, body mass index; AFC, antral follicle count; PID, pelvic inflammatory disease.

**Table 2:** IVF/ICSI cycle features in the three groups.

	SEP	REP	IUP	P-value ^a^		
	(n = 912)	(n = 457)	(n = 1169)	P1	P2	P3
No. of oocytes retrieved	6 (1–35)	7 (1–34)	8 (1–36)	0.242	0.000	0.002
Methods of fertilization (%)						
IVF	712 (78.1)	371 (81.2)	944 (80.8)	0.182	0.132	0.843
ICSI	200 (21.9)	86 (18.8)	225 (19.2)			
Normal fertilization rate	0.70 (0.07–1)	0.73 (0.08–1)	0.69 (0.07–1)	0.085	0.921	0.057
Stage of embryos transferred (%)						
Cleavage	672 (73.7)	319 (69.8)	793 (67.8)	0.130	0.004	0.443
Blastocyst	240 (26.3)	138 (30.2)	376 (32.2)			
Type of embryo transfer (%)						
Fresh	250 (27.4)	137 (30.0)	374 (32.0)	0.320	0.024	0.431
Frozen-thawed	662 (72.6)	320 (70.0)	795 (68.0)			
No. of embryos transferred (%)						
1	312 (34.2)	176 (38.5)	501 (42.9)	0.117	0.000	0.110
2	600 (65.8)	281 (61.5)	668 (57.1)			

*Note*: Values as median (range) or number (%).

^a^We defined the statistical outcomes of SEP group versus REP group as P1; SEP group versus IUP group as P2; REP group versus IUP group as P3.

Abbreviations: SEP, single ectopic pregnancy; REP, recurrent ectopic pregnancy; IUP, intrauterine pregnancy; IVF, in vitro fertilization; ICSI, intracytoplasmic sperm injection.

Pregnancy outcomes of each cohort are summarized in Table 3. The incidence of EP in the REP group after IVF treatment was significantly lower than those in the SEP group (2.4% vs. 6.8%, P = 0.011), and similar to those in the IUP group (2.4% vs. 2.1%, P = 0.830). There were no significant differences in the clinical pregnancy rate, miscarriage rate, and live birth rate among the three groups. Pregnancy outcomes of each cohort were stratified into the following categories according to maternal age: < 30 years, 30–35 years, and ≥ 35 years. In the subgroups of women aged 30–35 years and ≥ 35 years, women with previous SEP had significant higher rate of EP than those with previous IUP (6.4% vs. 1.1%, P = 0.002; 7.5% vs. 2.5%, P = 0.027, respectively). In the SEP group, 33 cases after embryo transfer had recurrent EP, including two patients with heterotopic pregnancy, 27 patients with tubal pregnancy, one patient with cornual pregnancy, and three patients with cesarean scar pregnancy. In the REP group, 3 cases with recurrent EP after IVF were tube EP, and 3 cases with tube stump. In the IUP group, ectopic pregnancy occurred in 14 cases after embryo transfer, including 12 cases of tubal pregnancy, one case of abdominal pregnancy, and one case of cesarean scar pregnancy.

**Table 3:** Pregnancy outcomes after IVF/ICSI, stratified by maternal age.

	SEP	REP	IUP	P-value ^a^		
	(n = 912)	(n = 457)	(n = 1169)			
				P1	P2	P3
Clinical pregnancy rate (%)						
Full cohort	485 (53.2)	252 (55.1)	652 (55.8)	0.492	0.238	0.818
< 30 y	135 (53.6)	82 (59.0)	181 (57.8)	0.302	0.311	0.817
30 to < 35 y	203 (58.3)	93 (54.7)	269 (56.6)	0.433	0.626	0.664
≥ 35y	147 (47.1)	78 (52.7)	202 (53.0)	0.263	0.122	0.948
Miscarriage rate (%)						
Full cohort	58 (12.0)	26 (10.3)	71 (10.9)	0.506	0.574	0.803
< 30 y	14 (10.4)	9 (11.0)	24 (13.38)	0.888	0.435	0.604
30 to < 35 y	27 (13.3)	7 (7.5)	29 (10.8)	0.148	0.402	0.366
≥ 35y	17 (11.6)	10 (12.8)	18 (8.9)	0.783	0.415	0.328
Live birth rate (%)						
Full cohort	416 (45.6)	220 (48.1)	568 (48.6)	0.377	0.177	0.871
< 30 y	119 (47.2)	72 (51.8)	150 (47.9)	0.386	0.868	0.447
30 to < 35 y	171 (49.1)	82 (48.2)	240 (50.5)	0.847	0.694	0.608
≥ 35y	126 (40.4)	66 (44.6)	177 (46.5)	0.392	0.109	0.700
Ectopic pregnancy rate (%)						
Full cohort	33 (6.8)	6 (2.4)	14 (2.1)	0.011	0.000	0.830
< 30 y	9 (6.7)	4 (4.9)	6 (3.3)	0.808	0.166	0.790
30 to < 35 y	13 (6.4)	1 (1.1)	3 (1.1)	0.087	0.002	0.975
≥ 35y	11 (7.5)	1 (1.3)	5 (2.5)	0.097	0.027	0.537

*Note*: Values as number (%).

^a^ We defined the statistical outcomes of SEP group versus REP group as P1; SEP group versus IUP group as P2; REP group versus IUP group as P3.

Abbreviations: SEP, single ectopic pregnancy; REP, recurrent ectopic pregnancy; IUP, intrauterine pregnancy; IVF, in vitro fertilization; ICSI, intracytoplasmic sperm injection.

To evaluate the effect of different treatments of previous ectopic pregnancy on the recurrence risk of EP, the patients were categorized into two groups: the salpingectomy group and the salpingostomy group. In the group with a single history of EP, the incidence of recurrent EP was 6.2% for salpingectomy treatment and 8.4% for salpingostomy, and in the previous REP group, 2.3% for salpingectomy and 4.4% for salpingostomy. There was no statistically significant difference in the recurrent EP rate between the two groups. Detailed results are shown in Table 4.

**Table 4:** Effect of previous ectopic pregnancy treatments on the incidence of ectopic pregnancy after IVF/ICSI.

	SEP	REP ^a^	P-value
Salpingectomy			
Ectopic pregnancy rate (%)	22/354 (6.2)	3/131 (2.3)	0.083
Salpingostomy			
Ectopic pregnancy rate (%)	11/131 (8.4)	2/45 (4.4)	0.382
P-value	0.397	0.818	

*Note*: Values as number (%).

^a^Seventy-six patients with clinical pregnancy were excluded because they had experienced both salpingectomy and salpingostomy treatments in their multiple EP conditions.

Abbreviations: SEP, single ectopic pregnancy; REP, recurrent ectopic pregnancy; IVF, in vitro fertilization; ICSI, intracytoplasmic sperm injection.

The ORs with 95% CIs of live birth following REP versus the other two types of pregnancy histories are presented in Table 5. Adjusting for female age, male factor, stimulation protocol, stage of embryos transferred, type of embryos transferred and the number of embryos transferred did not influence live birth rates for women with previous REP compared with women with previous SEP and those with IUP. As shown in Table 6, after adjusting for maternal and treatment factors that might influence EP, the results indicate that the odds of EP were 82.2% lower (OR 0.178, 95% CI 0.042–0.762; P = 0.020) in women who had blastocyst transfer compared with cleavage embryo transfer in SEP group. The odds of EP were over six times (OR 6.260, 95% CI 1.255–31.220; P = 0.025) in women who underwent double embryo transfer as opposed to single embryo transfer in the IUP group.

**Table 5:** Crude and adjusted odds ratios (ORs) of live birth after REP versus other pregnancy histories.

	Crude OR (95% CI)	Adjusted OR (95% CI)
<30 y		
REP versus IUP	1.078 (0.822–1.318)	1.808 (0.395–8.265)
REP versus SEP	0.592 (0.389–0.900)	0.818 (0.533–1.253)
30 to < 35 y		
REP versus IUP	0.971 (0.815–1.157)	0.926 (0.775–1.107)
REP versus SEP	1.022 (0.708–1.475)	0.930 (0.636–1.122)
≥ 35 y		
REP versus IUP	0.953 (0.788–1.153)	0.986 (0.806–1.206)
REP versus SEP	0.847 (0.572–1.255)	0.980 (0.807–1.190)

Abbreviations: SEP, single ectopic pregnancy; REP, recurrent ectopic pregnancy; IUP, intrauterine pregnancy; CI, confidence interval.

**Table 6:** Risk factors associated with EP by logistic regression analysis.

Factor	Adjusted OR (95% CI)		
	SEP	REP	IUP
Female age			
< 30	REF	REF	REF
30 to < 35	1.758(0.711–4.348)	1.586(0.246–10.218)	1.294(0.382–4.381)
≥ 35	1.490(0.623–3.562)	0.444(0.039–5.078)	0.477(0.111–2.043)
Male factor			
Yes	REF	REF	REF
No	0.936(0.378–2.320)	1.540(0.265–8.963)	1.631(0.529–5.030)
Stimulation protocol			
Long protocol	REF	REF	REF
Short protocol	0.533(0.198–1.436)	0.264(0.016–4.398)	0.306(0.049–1.895)
Micro-stimulation protocol	0.819(0.272–2.463)	2.274(0.223–23.186)	0.630(0.084–4.699)
Others	0.517(0.152–1.761)	0.669(0.040–11.234)	1.820(0.360–9.190)
Stage of embryos transferred			
Cleavage	REF	REF	REF
Blastocyst	0.178(0.042–0.762) ^$^	0.386(0.037–3.998)	2.466(0.764–7.961)
Type of embryo transfer			
Fresh	REF	REF	REF
Frozen-thawed	1.195(0.503–2.835)	0.591(0.096–3.640)	0.670(0.208–2.160)
No. of embryos transferred			
1	REF	REF	REF
2	1.352(0.592–3.090)	1.081(0.179–6.511)	6.260(1.255–31.220) *

^$^Statistically significant (P = 0.020).

*Statistically significant (P = 0.025).

Abbreviations: SEP, single ectopic pregnancy; REP, recurrent ectopic pregnancy; IUP, intrauterine pregnancy; CI, confidence interval; REF, reference.

## Discussion

To our knowledge, our study is the first time to report clinical outcomes of IVF treatment in infertile women with previous recurrent EP and to evaluate the influence of different surgical methods on the probability of EP. The overall EP rate for women with previous single EP and recurrent EP (5.3%) in this study was consistent with that reported previously (5.1%) [7]. Interestingly, women who had a history of recurrent EP had a lower recurrence risk of EP compared with women with a history of single EP. Previous single EP significantly increased the risk of recurrent EP. The differences in the clinical pregnancy rate, miscarriage, and live birth rate among the three groups were not statistically significant.

To date, no in-depth comparison of the clinical outcomes of patients with recurrent EP to those with a single EP or IUP has been performed. Our data show that the incidence of EP was 2.4% for women with previous REP, which was similar to women with previous IUP (2.1%), but significantly lower than those with previous single EP (6.8%). It is not clear why patients with REP have a decreased risk of EP, but several factors could contribute. First, we speculate that repeat surgical treatments of EPs may reduce the risk of EP. According to a newly published study reported by Karavani G et al., surgical intervention of a second EP could significantly decrease the risk of a third EP [14]. We have not found other studies assessing this risk of a history of multiple EPs in a controlled design. Second, infertility duration for women with single EP was significantly longer compared with women with recurrent EP in this study. This implies that a long attempt time to pregnant may play a role in the etiology of EP occurrence [16].

Current evidence supports the hypothesis that tubal EP is resulted from embryo retention within the tube caused by altered embryo-tubal transport and change in the tubal environment [17]. In published studies, the risk factors for tubal EP are well established and include: tubal disorders as a result of surgery or infection, ART technologies, and smoking [17]. Tubal disorders have been regarded as a major risk factor for EP, accounting for a third of all cases [18]. It has been shown that among women with a history of EP, the risk of recurrent EP is approximately five to ten times higher than those without a history of EP [9, 18]. In this study, the rate of EP in women with SEP was 6.8%, significantly higher than that in the control group (2.1%). It is possible because in women with tubal EP, surgery remains the treatment of first choice [19]. The resulting damage to the tubes may cause the alteration of the embryo-tubal transport and tubal environment, and is, therefore, one of the major reasons to raise the incidence of recurrent EP.

EP after IVF is comparable to or even more common than natural pregnancy [4–6], although IVF allows embryos to be transferred directly into an accurate area in the uterine cavity and completely bypasses the fallopian tube. The exact reason why the transferred embryos migrate from the uterine cavity to implant extrauterine remains unclear. The type of embryos transferred [10], the stage of embryos transferred [5, 10], and the technique of embryo transfer [20–22] are potential causes but there is little evidence to support these. Women who receive stimulated cycles are at higher risk of tubal ectopic pregnancy compared with women who undergo natural cycles [5, 23]. In addition, the cell adhesion protein, E-cadherin is associated with the occurrence of ectopic pregnancy [24]. Revel et al. reported that the expression of E-cadherin at the tubal implantation sites in women with an IVF pregnancy was stronger than that in spontaneous tubal pregnancies [24], which suggested a different mechanism of ectopic pregnancy. Bhattacharya et al. showed that a history of previous EP was an important risk factor for recurrent EP [25]. Our results showed that patients with a single history of EP had a higher incidence of EP compared with women with no history of EP. Contrary to our finding, the study reported by Cai H et al. reported that previous tubal EP did not raise the incidence of recurrent EP in IVF [8]. The reason may be partially due to the difference in the study population and only patients with tubal factor infertility were included in the study of Cai H et al.. Additionally, we speculate that another reason for the higher recurrent EP rate in our study could be explained by the higher proportion of cleavage embryo transferred cycles, as it is suggested that blastocyst transfer may reduce the incidence of EP [26, 27]. Theoretically, due to having a larger diameter and shorter time before implantation, blastocyst transfer would be expected to have a higher likelihood of intrauterine implantation and a lower likelihood of ectopic implantation, than cleavage-stage embryo transfer [26, 27].

In the present study, the number of embryos transferred was associated with the risk of EP after adjusting for multiple factors. It was observed that the number of embryos transferred is an important underlying factor in the aetiology of EP after IVF treatment [28]. A similar finding reported by Yanaihara et al. showed that the likelihood of EP was significantly lower with single embryo transfer than with double embryo transfer [29]. In addition, a retrospective study found that transfer with fewer embryos may contribute to decreasing the rate of EP [5]. However, a meta-analysis suggested that the EP rate for elective single embryo transfer was not statistically significantly different from that for double embryo transfer (relative risk 0.42, 95% CI 0.09–2.01) [30]. This may be due to the small sample size, in that there were only six ectopic pregnancies reported in the three studies included in the meta-analysis [30].

Similar to previous reports [23, 31], the risk of EP in the REP and IUP groups has a decreasing trend after frozen-thawed embryo transfers than after fresh embryo transfer. Some hypotheses have been proposed to explain the increased risk of EP in stimulated cycles; these include an altered uterine environment or endometrial receptivity that may have a negative effect on embryo-tubal transport [23, 31], and the oocyte retrieval procedure that could lead to uterine contractility and the release of inflammation mediators adjacent to the fallopian tube [4].

Women with IUP had a significantly higher number of oocytes retrieved than those in the SEP and REP groups. The result may be explained by the different ovarian responses to stimulation. Evidence suggested that AFC was a marker of ovarian reserve and may be helpful in predicting the number of oocytes retrieved and stimulation response [32]. We collected and calculated AFC at the beginning of the cycle and found that women in the IUP group had a significantly higher AFC than those with SEP and REP. This finding could be explained by the effect of tubal surgery for EP on ovarian reserve and ovarian response. Because ovarian vascularization is close to the fallopian tube and mesosalpinx, tubal surgery may impair ovarian vascularization and function [33, 34].

Tubal EP can be surgically managed by salpingectomy, in which the affected tube is removed, or salpingostomy, in which the tube is conserved and only the trophoblast is removed [35]. Several investigations have attempted to evaluate future fertility prospects after different surgical approaches of tubal EP [7, 36]. In a randomized controlled trial, the repeat ectopic pregnancy rate was 8% after salpingostomy and 5% after salpingectomy (P = 0.19) [36]. In 2015 a large retrospective study showed that the rate of ectopic pregnancy after IVF was 4.9% in the salpingostomy group, which was similar to that in the salpingectomy group (4.5%) [7]. Consistent with these studies [7, 36], our findings show that there is no significant difference between salpingostomy and salpingectomy treatments. Additionally, in this study, for patients who were treated with salpingectomy, the ectopic pregnancy rate (6.2%) in the SEP group was slightly higher than that of the REP group (2.3%), but the difference was not statistically significant (P = 0.083). The occurrence of ectopic pregnancy after bilateral salpingectomy is rare. The incidence of spontaneous pregnancy after bilateral salpingectomy is very low. With the wide application of IVF-ET, successful pregnancy after bilateral salpingectomy can be achieved, but it also increases the risk of ectopic pregnancy [37, 38]. A recent review summarized that the sites of ectopic pregnancy following IVF-ET after bilateral salpingectomy include tube stump, uterine cornua, abdominal cavity, retroperitoneum, and ovaries [39]. In this study, three patients after bilateral salpingectomy developed ectopic pregnancy following IVF-ET. The sites of ectopic pregnancy were all tubal stumps.

Our study is valuable because this is the first to provide evidence for the effect of a history of recurrent EP on outcomes of subsequent IVF treatment. This is also the largest study of a population with previous recurrent EP. However, our study has several limitations. First, due to the retrospective nature of the study, selection bias derived from the use of medical records and patient report may be inevitable. When the treatment for EP was not managed in our hospital, the information was based on the patient’s report during the IVF cycle. This is the second limitation of the study. Finally, we cannot exclude unmeasured confounding as an explanation of our findings. For instance, we did not consider the use of intrauterine devices or condoms which may be a significant risk factor for recurrent EP [18]. Despite these limitations, an important strength that enhances the validity of our data is the consistency of a single-center experience in clinical and laboratory protocols (e.g., ovarian stimulation, embryo transfer technique, embryo culture system, embryo cryopreservation, and thaw protocols). Our findings add information to the limited available data about reproductive outcomes of IVF treatment for women with a history of recurrent EP.

## Conclusions

Women with previous recurrent EP had lower risks of EP after IVF treatment compared with women with one history of EP. Previous single EP significantly increased the risk of recurrent EP. No significant differences were observed in the clinical pregnancy rate, miscarriage rate, and live birth rate among the three groups. The salpingostomy and salpingectomy treatments of EP did not influence the incidence of recurrent EP after IVF. The results suggest that closer attention to the EP history of patients undergoing IVF is warranted.

## Supporting information

S1 Data (XLSX)
